# Moroccan women and tattoos: characteristics, perception and stigmatization

**DOI:** 10.1192/j.eurpsy.2026.11449

**Published:** 2026-07-31

**Authors:** A. Souidi, G. Hami, S. Kailil, S. Stati, S. Belbachir, A. Ouanass

**Affiliations:** Ar-razi, Rabat, Morocco

## Abstract

**Introduction:**

Tattooing is a mode of bodily expression that belongs to both tradition and modernity. This form of expression is rich in variant and variable meanings for women in general, and women with psychiatric disorders in particular. Their popularity and prevalence are increasing rapidly, particularly among young people. However, despite this expansion, the social perception of tattoos, particularly among women, remains a complex and often stigmatized subject. Hence the interest in knowing the characteristics of tattoos among women in Morocco, the different perceptions of the latter as well as the correlation of tattooing with psychiatric disorders.

**Objectives:**

To study the characteristics, meaning and perception of tattoos among Moroccan women, past and present. In an attempt to better understand the potential health implications of this mode of expression and its correlation with mental health.

**Methods:**

A descriptive cross-sectional study was carried out among women of different profiles. Data were collected using a form sent online and distributed in paper form. The form collected socio-demographic data, descriptive characteristics of the tattoos and the women’s mental health status. Statistical analysis was performed using Jamovi 2.3.

**Results:**

Our results focused on the socio-demographic characteristics of our population, it was carried out on 52 women, 57.7% of whom were aged between 25 and 40, 62% of our sample were single and the majority(92%) had a level of education higher than the baccalaureate. Also, almost 30% were unemployed.

ours study results focused essentially on the tattoo characteristics, summarized in Image 1 . Image 2 shows the results concerning the psychiatric history of our population.

**Image 1:**
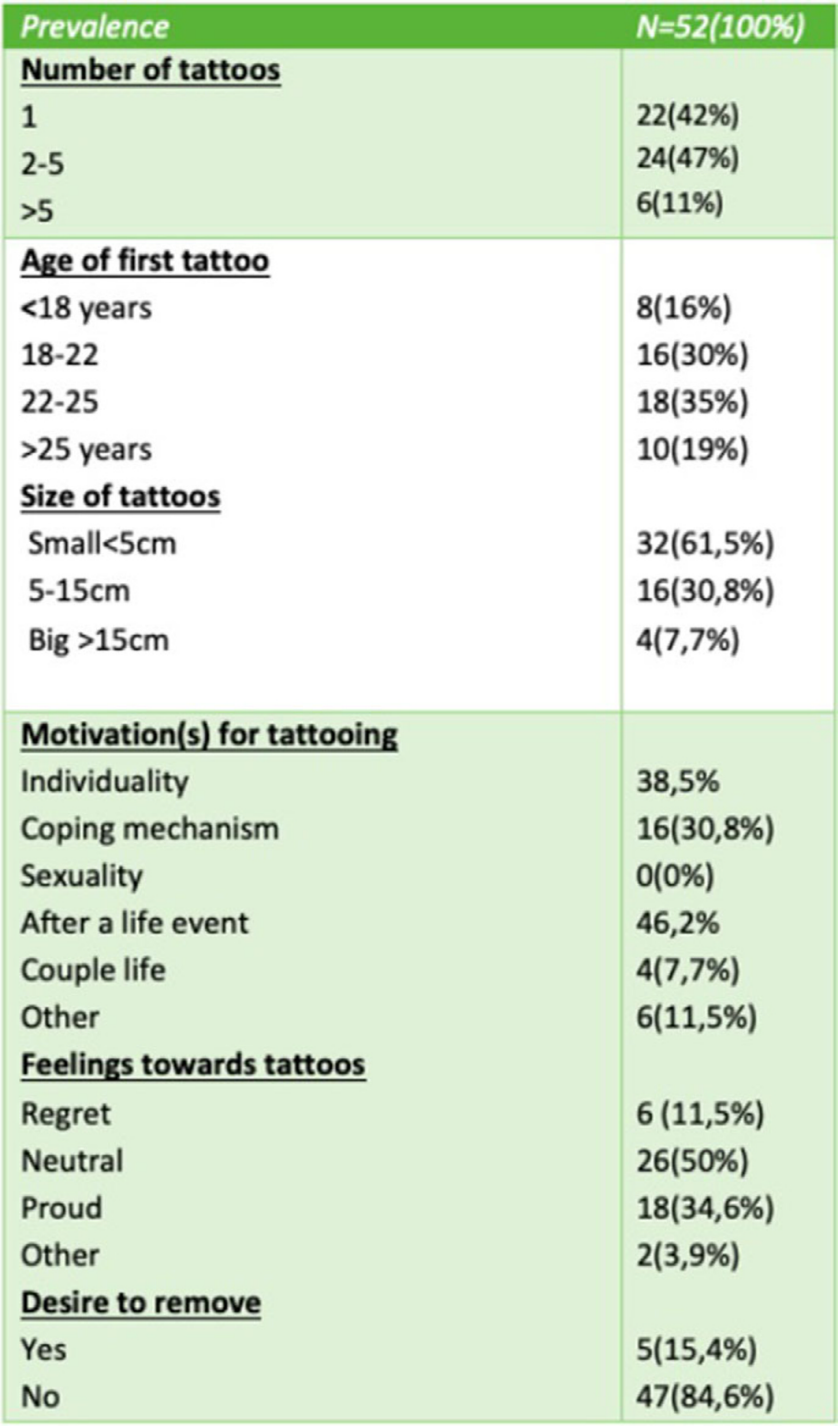
Image 1: Long description.

**Image 2:**
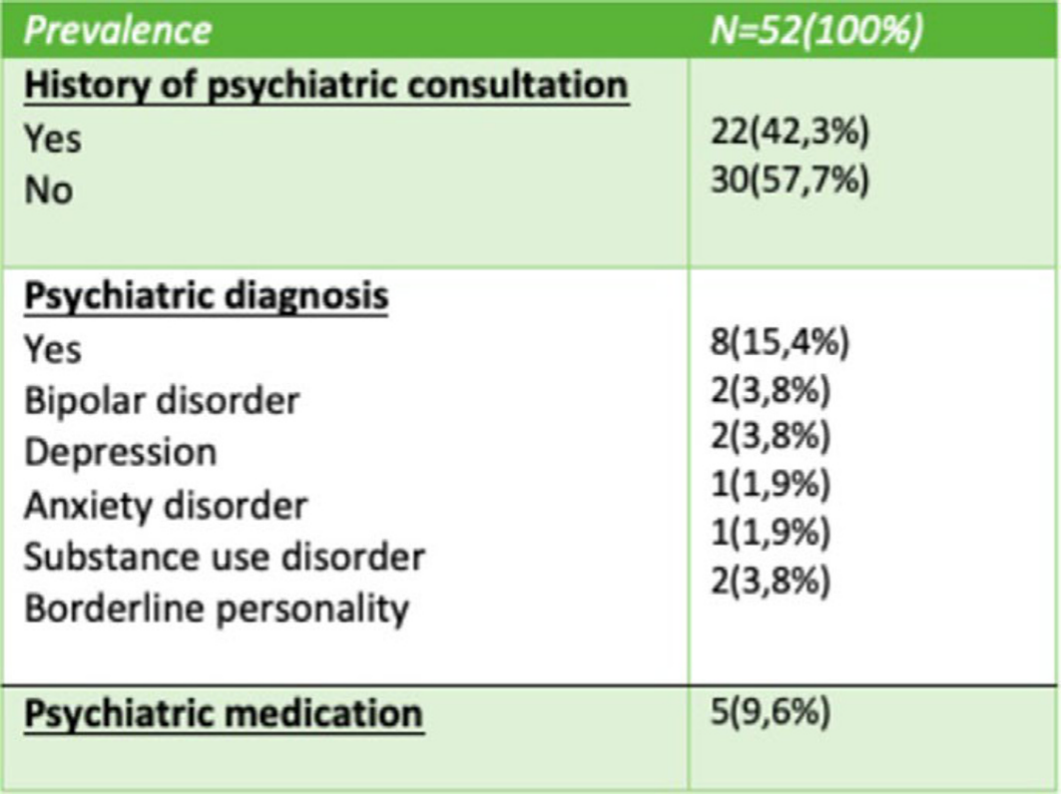
Image 2: Long description.

**Conclusions:**

Tattooing is a means of expression and a form of body modification, rich in cultural and personal significance, with a dual affiliation to tradition and modernity. It is highly prevalent among women, and has implications for both mental and physical health.

**Disclosure of Interest:**

None Declared

## Long descriptions

### Image 1: Long description

The table is titled Prevalence with a total sample size N equals 52 (100%). It is divided into six sections.

1. Number of tattoos

- 1: 22 (42%)

- 2-5: 24 (47%)

- >5: 6 (11%)

2. Age of first tattoo

- <18 years: 8 (16%)

- 18-22: 16 (30%)

- 22-25: 18 (35%)

- >25 years: 10 (19%)

3. Size of tattoos

- Small <5cm: 32 (61,5%)

- 5-15cm: 16 (30,8%)

- Big >15cm: 4 (7,7%)

4. Motivation(s) for tattooing

- Individuality: 38,5%

- Coping mechanism: 16 (30,8%)

- Sexuality: 0 (0%)

- After a life event: 46,2%

- Couple life: 4 (7,7%)

- Other: 6 (11,5%)

5. Feelings towards tattoos

- Regret: 6 (11,5%)

- Neutral: 26 (50%)

- Proud: 18 (34,6%)

- Other: 2 (3,9%)

6. Desire to remove

- Yes: 5 (15,4%)

- No: 47 (84,6%)

Navigate back to Image 1:.

### Image 2: Long description

The table is divided into two columns. The left column lists categories and the right column lists the count and percentage where N equals 52 (100 percent).

1. History of psychiatric consultation

- Yes: 22 (42,3 percent)

- No: 30 (57,7 percent)

2. Psychiatric diagnosis

- Yes: 8 (15,4 percent)

- Bipolar disorder: 2 (3,8 percent)

- Depression: 2 (3,8 percent)

- Anxiety disorder: 1 (1,9 percent)

- Substance use disorder: 1 (1,9 percent)

- Borderline personality: 2 (3,8 percent)

3. Psychiatric medication

- Total: 5 (9,6 percent)

Navigate back to Image 2:.

